# Acceptance of emerging renal oncocytic neoplasms: a survey of urologic pathologists

**DOI:** 10.1007/s00428-024-03909-2

**Published:** 2024-09-17

**Authors:** Sambit K. Mohanty, Anandi Lobo, Shilpy Jha, Ankur R. Sangoi, Mahmut Akgul, Kiril Trpkov, Ondrej Hes, Rohit Mehra, Michelle S. Hirsch, Holger Moch, Steven C. Smith, Rajal B. Shah, Liang Cheng, Mahul B. Amin, Jonathan I. Epstein, Anil V. Parwani, Brett Delahunt, Sangeeta Desai, Christopher G. Przybycin, Claudia Manini, Daniel J. Luthringer, Deepika Sirohi, Deepika Jain, Divya Midha, Ekta Jain, Fiona Maclean, Giovanna A. Giannico, Gladell P. Paner, Guido Martignoni, Hikmat A. Al-Ahmadie, Jesse McKenney, John R. Srigley, Jose Ignacio Lopez, L. Priya Kunju, Lisa Browning, Manju Aron, Maria M. Picken, Maria Tretiakova, Ming Zhou, Mukund Sable, Naoto Kuroda, Niharika Pattnaik, Nilesh S. Gupta, Priya Rao, Samson W. Fine, Pritinanda Mishra, Amit K. Adhya, Bijal N. Kulkarni, Mallika Dixit, Manas R. Baisakh, Samriti Arora, Sankalp Sancheti, Santosh Menon, Sara E. Wobker, Satish K. Tickoo, Seema Kaushal, Shailesh Soni, Shivani Kandukuri, Shivani Sharma, Suvradeep Mitra, Victor E. Reuter, Vipra Malik, Vishal Rao, Ying-Bei Chen, Sean R. Williamson

**Affiliations:** 1 Department of Pathology, Advanced Medical Research Institute Hospital, Kolkata, India; 2 Department of Pathology, Core Diagnostics, Gurgaon, India; 3 Department of Pathology, Kapoor Center of Urology and Pathology, Raipur, India; 4 Department of Pathology, El Camino Hospital, Mountain View, USA; 5 Department of Pathology, Albany Medical Centre, Albany, USA; 6 Department of Pathology, University of Calgary, Calgary, Canada; 7 Department of Pathology, Bioptika Laborator S.R.O, Pilsen, Czech Republic; 8 Department of Pathology, University of Michigan, Ann Arbor, USA; 9 Department of Pathology, Brigham and Women’s Hospital, Boston, USA; 10 Department of Pathology, University Hospital, Zurich, Switzerland; 11 Department of Pathology, Virginia Commonwealth University School of Medicine, Richmond, USA; 12 Department of Pathology, UT Southwestern Medical Center, Dallas, USA; 13 Department of Pathology, Brown University, Providence, USA; 14 Department of Pathology, The University of Tennessee Health Science Center, Memphis, USA; 15 IMP Pathology, Garden City, USA; 16 Department of Pathology, Ohio State University, Columbus, USA; 17 Department of Pathology, Wellington School/Medicine, Wellington, New Zealand; 18 Department of Pathology, Tata Memorial Hospital, Mumbai, India; 19 Department of Pathology, Cleveland Clinic, Cleveland, USA; 20 Department of Pathology, University of Turin, Turin, Italy; 21 Department of Pathology, Cedars-Sinai Medical Center, Los Angeles, USA; 22 Department of Pathology, University of Utah/ARUP, Salt Lake City, USA; 23 Department of Pathology, Tata Medical Center, Kolkata, India; 24 Department of Pathology, Douglass Hanly Moir Pathology, Sydney, Australia; 25 Department of Pathology, Vanderbilt University Medical Center, Nashville, USA; 26 Department of Pathology, University of Chicago, Chicago, USA; 27 Department of Pathology, University of Verona, Verona, Italy; 28 Department of Pathology, Memorial Sloan Kettering Cancer Center, New York, USA; 29 Department of Pathology, Trillium Health Partners, Credit Valley Hospital, Mississauga, Canada; 30 Department of Pathology, Cruces University Hospital, Barakaldo, Spain; 31 Department of Pathology, Oxford University Hospital NHS Foundation Trust, Oxford, UK; 32 Department of Pathology, Keck School of Medicine of USC, Los Angeles, USA; 33 Department of Pathology, Loyola University Medical Center, Maywood, USA; 34 Department of Pathology, University of Washington, Seattle, USA; 35 Department of Pathology, Tufts University School of Medicine, Boston, USA; 36 Department of Pathology, All India Institute of Medical Sciences, Bhubaneswar, India; 37 Department of Pathology, Kochi Red Cross Hospital, Kochi City, Kochi Japan; 38 Department of Pathology, Henry Ford Health System, Detroit, USA; 39 Department of Pathology, The University of Texas MD Anderson Cancer Center, Houston, USA; 40 Department of Pathology, Kokilaben Ambani Hospital, Mumbai, India; 41 Department of Pathology, Apollo Hospitals Bhubaneshwar, Bhubaneswar, India; 42 Department of Pathology, Homi Bhabha Cancer Center, Visakhapatnam, India; 43 Department of Pathology, The University of North Carolina at Chapel Hill, Chapel Hill, USA; 44 Department of Pathology, All India Institute of Medical Sciences, New Delhi, India; 45 Department of Pathology, Muljibhai Patel Urological Hospital, Nadiad, India; 46 Department of Pathology, Postgraduate Institute of Medical Education and Research, Chandigarh, India; 47 Department of Pathology, Basavatakaram Indo-American Cancer Hospital and Research Institute, Hyderabad, India

**Keywords:** Eosinophilic, Oncocytic, Renal neoplasms, Emerging, Uropathologists

## Abstract

Oncocytic renal neoplasms are a major source of diagnostic challenge in genitourinary pathology; however, they are typically nonaggressive in general, raising the question of whether distinguishing different subtypes, including emerging entities, is necessary. Emerging entities recently described include eosinophilic solid and cystic renal cell carcinoma (ESC RCC), low-grade oncocytic tumor (LOT), eosinophilic vacuolated tumor (EVT), and papillary renal neoplasm with reverse polarity (PRNRP). A survey was shared among 65 urologic pathologists using SurveyMonkey.com (Survey Monkey, Santa Clara, CA, USA). De-identified and anonymized respondent data were analyzed. Sixty-three participants completed the survey and contributed to the study. Participants were from Asia (*n* = 21; 35%), North America (*n* = 31; 52%), Europe (*n* = 6; 10%), and Australia (*n* = 2; 3%). Half encounter oncocytic renal neoplasms that are difficult to classify monthly or more frequently. Most (70%) indicated that there is enough evidence to consider ESC RCC as a distinct entity now, whereas there was less certainty for LOT (27%), EVT (29%), and PRNRP (37%). However, when combining the responses for sufficient evidence currently and likely in the future, LOT and EVT yielded > 70% and > 60% for PRNRP. Most (60%) would not render an outright diagnosis of oncocytoma on needle core biopsy. There was a dichotomy in the routine use of immunohistochemistry (IHC) in the evaluation of oncocytoma (yes = 52%; no = 48%). The most utilized IHC markers included keratin 7 and 20, KIT, AMACR, PAX8, CA9, melan A, succinate dehydrogenase (SDH)B, and fumarate hydratase (FH). Genetic techniques used included *TSC1/TSC2/MTOR* (67%) or *TFE3* (74%) genes and pathways; however, the majority reported using these very rarely. Only 40% have encountered low-grade oncocytic renal neoplasms that are deficient for FH. Increasing experience with the spectrum of oncocytic renal neoplasms will likely yield further insights into the most appropriate work-up, classification, and clinical management for these entities.

**Supplementary Information:**

The online version contains supplementary material available at 10.1007/s00428-024-03909-2.

## Introduction

Oncocytic renal neoplasms are a recurrent source of diagnostic difficulty in urologic pathology; however, their behavior is largely favorable, raising the question of whether subdividing into multiple diagnostic entities is important. Nonetheless, several emerging diagnostic entities composed of eosinophilic cells have been recently characterized, several of which are not yet included in the WHO Classification. These have reproducible histologic, immunohistochemical, and molecular features, such as eosinophilic solid and cystic renal cell carcinoma (ESC RCC, a WHO entity), low-grade oncocytic renal tumor (LOT, not yet a WHO entity), and eosinophilic vacuolated tumor (EVT, previously reported as high-grade oncocytic tumor/RCC with eosinophilic vacuolated cytoplasm, not yet a WHO entity) [1, 2]. Awareness and acceptance of these entities among expert uropathologists and uro-oncologists are still evolving and debated. A recent editorial suggested that such subdivision of eosinophilic renal tumors is unnecessary and not clinically relevant [3]. This controversy prompted us to undertake a multi-institutional and international survey to assess reporting trends, practices, and resource utilization in the oncocytic tumors among uropathologists.

## Material and methods

This study was conducted after approval from the institutional review board. A questionnaire and scenario-based survey were sent to a broad group of genitourinary (GU) pathologists with questions related to the morphologic, IHC, and molecular parameters of oncocytic renal neoplasms. The online survey containing 27 questions was prepared by three of the authors (SRW, SKM, and AL) and circulated among 65 GU Pathologists in four continents in September 2021 (Supplement). Questions included currently accepted/preferred terminology, frequency of encountering these neoplasm(s), and preferred testing techniques. The study was carried out in accordance with The Code of Ethics of the World Medical Association (Declaration of Helsinki). Informed consent was obtained from the pathologists through e-mails, and the intended use of the data was explained. The surveyed pathologists were given the option of authorship at the onset of the survey and were given the option to withdraw participation at any time including at the completion of the survey or afterward. Each participant was grouped based on their practicing experiences in years as < 5 years, 5–10 years, 10–20 years, and > 20 years. Analyses of survey responses were carried out using the SurveyMonkey software (http://www.surveymonkey.com; SurveyMonkey, Santa Clara, CA, USA). A multiple-choice format was utilized. Respondents were asked to choose the best possible answer based on their practice and recommended protocols. De-identified and anonymized respondent data were tabulated and analyzed utilizing descriptive statistics.

## Results

Sixty-three (97%) participants completed the survey and were included in the study including the three survey authors (SKM, SRW, and AL). Six (10%), 11 (18%), 28 (44%), and 18 (29%) participants had < 5 years, 5–10 years, 10–20 years, and > 20 years of experience reporting GU pathology specimens, respectively (Question #1). Participants represented Asia (*n* = 21; 33%), North America (*n* = 34; 54%), Europe (*n* = 6; 10%), and Australia (*n* = 2; 3%) (Question #2). Among participating uropathologists, eosinophilic renal neoplasms on average made up 5–20% of tumors received in their practice. A few participants indicated that as much as 50% of their renal tumor specimens were eosinophilic (Question #3). Respondents reported that as many as 60% of these tumors could be definitively classified as belonging to a specific subtype (Question #4). About half of participating uropathologists (*n* = 30; 48%) encounter a difficult-to-classify eosinophilic/oncocytic renal neoplasm approximately monthly, whereas 15 (24%) came across such neoplasms every few months, and 12 (19%) a few times in a year (Question #5).

At the time of the survey, thirty-two (51%) participants chose the response that there was currently not enough evidence to regard LOT (keratin 7 + /KIT −) as a distinct entity but likely would be eventually. In contrast, 17 (27%) felt that there was enough evidence to regard LOT as an independent entity, and 8 (13%) were of the opinion that this tumor can be grouped with one or more other emerging oncocytic entities; the remainder were either uncertain (*n* = 4; 6%) or felt that it is not a distinct entity (*n* = 2; 3%) (Question #6). Similarly, when asked the same question regarding acceptance of EVT as a distinct tumor entity, the responses at the time of the survey were 28 (44%) responded that there is not sufficient evidence currently but likely would be eventually. In contrast, 18 (29%) felt there was sufficient evidence for EVT as a definite tumor entity now, 9 (14%) believed that this tumor should be grouped with one or more other emerging oncocytic entities, and the remainder were either uncertain (*n* = 7; 11%) or felt that it is not a distinct entity (*n* = 1; 2%) (Question #7). For both LOT and EVT, when combining the responses indicating that there was sufficient evidence now for a distinct entity, or likely would be in the future, these both yielded greater than 70% for current or eventual consideration as a distinct tumor type.

For ESC RCC, 44 (70%) felt that there is enough evidence to accept it as a distinct independent tumor entity now; 7 (11%) would await more studies, 6 (10%) believed that this tumor should be grouped with one or more other emerging oncocytic entities, 5 (8%) were uncertain, and 1 (2%) felt that it is not a distinct entity (Question #8).

The uropathologists surveyed report encountering the aforementioned oncocytic tumors (LOT, EVT, and ESC RCC) daily/weekly (*n* = 7, 12%), few times a month/monthly (*n* = 15, 24%), every few months (*n* = 29; 46%), or yearly (*n* = 10; 16%); 2 reported never seeing such lesions (Question #9). Thirty-eight (60%) survey participants would never render an outright diagnosis of oncocytoma in a needle biopsy specimen, whereas 12 (19%) would. Thirteen (21%) would report a diagnosis favoring oncocytoma only if the features were typical, with concordant morphology and immunohistochemistry.

A subset of pathologists would favor using the terminology “low-grade renal oncocytic neoplasm, favoring oncocytoma” in case of concordant histomorphology and IHC, and a more equivocal terminology such as “renal oncocytic neoplasm, indeterminate/cannot exclude RCC” where morphology and IHC were discordant. If the patient had a history of BHD or prior oncocytoma/chromophobe, or multiple lesions, then the possibility of “hybrid oncocytoma chromophobe tumor” could be raised (Question #10). In the context of overlapping/conflicting morphological and immunohistochemical oncocytic/eosinophilic neoplasm on needle biopsy, the majority of the uropathologists (*n* = 57; 91%) concurred that the most appropriate diagnostic terminology would be “oncocytic neoplasm,” with an explanatory note regarding the overlapping findings (Question #11).

Most participating pathologists (*n* = 59; 94%) felt that it is mandatory to distinguish an oncocytoma from other oncocytic renal neoplasms (Question #12). Forty-two (67%) participants would render a diagnosis of an oncocytoma in an oncocytic tumor with classical oncocytoma morphology and diffuse positivity for KIT in the complete absence of keratin 7 staining; 14 (22%) would be cautious and stated that this statement would qualify only on a resection specimen and not on a core needle biopsy. Most of the pathologists noted that minimal staining for keratin 7 in a few scattered cells is expected in an oncocytoma, whereas a complete absent staining pattern was unusual (Question #13).

**Fig. 1:**
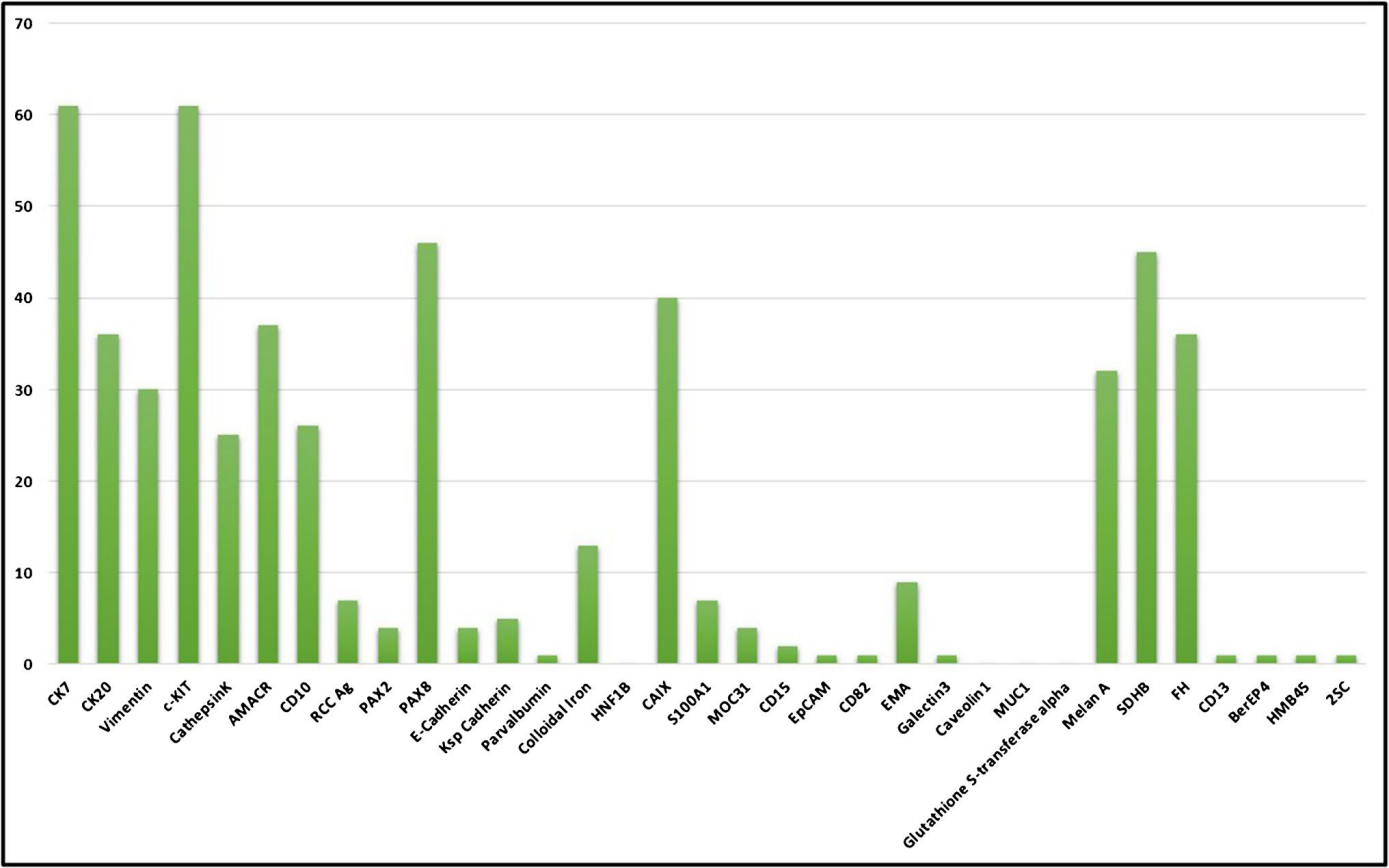
Bar graph depicting the immunohistochemical stains of choice used by the survey participants in the differential diagnosis of eosinophilic renal tumors

There was significant variability in the choice of IHC work up for oncocytic renal tumors. Among survey pathologists, the most widely used markers selected were keratin 7 (*n* = 61; 97%), KIT (*n* = 61; 97%), PAX8 (*n* = 46; 73%), SDHB (*n* = 45; 71%), CA9 (*n* = 40; 64%), AMACR (*n* = 37; 59%), keratin 20 (*n* = 36; 57%), FH (*n* = 36; 57%), melan A (*n* = 32; 51%), vimentin (*n* = 30; 48%), CD10 (*n* = 26; 41%), and cathepsin K (*n* = 25; 40%) (Question #14) (Fig. 1). Approximately one-half of uropathologists would routinely use IHC in the work up of oncocytoma (yes = 52%; no = 48%) (Question #15). Features that would lead respondents to perform IHC in a possible oncocytoma included mild nuclear membrane irregularity with subtle perinuclear clearing (*n* = 34; 54%), compact nesting/solid pattern (*n* = 23; 37%), vascular invasion (*n* = 20; 32%), fat invasion (*n* = 19; 31%), “small cell” or “oncoblastic” features (*n* = 16; 25%), nuclear grooves (*n* = 13; 21%), or binucleation (*n* = 12; 19%). A subset of 20 (32%) uropathologists reported that they would always use IHC in the diagnosis of an oncocytoma, regardless of the prior morphologic features, and another subset of 8 (13%) would not use IHC at all (Question #16).

Around half of the survey participants (*n* = 32; 51%) would utilize the terminology “low-grade oncocytic tumor (LOT)” for an oncocytic tumor diffusely positive for keratin 7 and negative for KIT; 12 (19%) would diagnose it as oncocytoma or chromophobe RCC, and another 14 (22%) would report these lesions along with a differential diagnosis and with a comment stating that while the morphology and IHC are similar to those that have been described for LOT, the tumor has not been fully characterized and patient outcome associated with these tumors may not yet be fully understood. A subset of pathologists still preferred labeling these lesions as “RCC-unclassified” or “unclassified low-grade oncocytic neoplasm” (Question #17).

In response to a case scenario, 21 (33%) pathologists would label a renal tumor as unclassifiable if it showed a mixture of eosinophilic papillary and non-papillary morphology without cystic areas and diffuse positivity for keratin 20, in which FH and SDHB are retained/normal within the tumor cells; 32 (51%) respondents would categorize such a tumor as ESC RCC (Question #18).

Regarding the routine use of cathepsin K IHC in the workup of oncocytic neoplasms: 23 (37%) reported using it only in certain scenarios to arrive at a specific diagnosis, 12 (19%) frequently use cathepsin K IHC in most oncocytic neoplasms, whereas 16 (25%) noted that it was not available in their area of practice (Question #19). A majority (*n* = 39; 62%) of participants perform TFE3 IHC, either frequently, or as needed, to arrive at a specific diagnosis (Question #20). Similarly, the majority (*n* = 39; 62%) would utilize TFE3 FISH/NGS in the diagnostic work up of an eosinophilic renal epithelial neoplasm, either frequently, or as needed, to arrive at a more definitive diagnosis (Question #21).

When asked about the most appropriate terminology for a tumor fitting the description of the recently-described entity eosinophilic vacuolated tumor (EVT)/high-grade oncocytic tumor/renal cell carcinoma with eosinophilic vacuolated cytoplasm, majority (*n* = 34; 54%) were in favor of the terminology “eosinophilic vacuolated tumor,” 10 (16%) supported the term “renal cell carcinoma with eosinophilic vacuolated cytoplasm,” and another 6 (10%) preferred the terminology “high-grade oncocytic tumor” (Question #22).

The most commonly applied genetic tests for the diagnosis of oncocytic renal neoplasms are as follows: TFE3/FISH (*n* = 45; 71%), TFEB/FISH (*n* = 31; 49%), TSC1/NGS (*n* = 21; 33%), TSC2/NGS (*n* = 20;32%), and MTOR/NGS (*n* = 17; 27%); however, over half of the survey participants replied that they would use genetic tests very rarely (*n* = 35; 56%) (Questions #23 and 24). Genetics were reported to be used in most/all oncocytic tumors by only 1 participant (2%), 25–50% of tumors by 8%, 25% of tumors or less by 24%, and never by 11%.

The majority of participating genitourinary pathologists (*n* = 44; 70%) would ask for familial screening for extrarenal neoplasms such as gastrointestinal stromal tumor in a subset of oncocytic renal neoplasms, particularly SDH-deficient RCC, whereas 19 (30%) would not (Question #25).

Regarding papillary renal neoplasm with reverse polarity (PRNRP)/oncocytic papillary renal neoplasm with inverted nuclei: 24 (38%) felt that there is enough evidence to accept PRNRP as a distinct independent tumor entity, 16 (25%) felt that there likely would be enough evidence in the future, 9 (14%) responded that this tumor should be grouped together with papillary renal carcinoma, whereas 13 (21%) were uncertain, and 1 (2%) felt that it is not a distinct entity (Question #26). Combining the responses of sufficient evidence now and likely in the future, this yields 63% who could be considered positive regarding the future incorporation of this entity into classification schemes.

Twenty-five (40%) participating uropathologists have encountered low-grade oncocytic renal neoplasms that are deficient for fumarate hydratase (FH, mimicking SDH-deficient renal cell carcinoma), typically within consultation practice, whereas 35 (56%) have not (Question #27).

## Discussion

Renal cell tumors composed of eosinophilic cells remain a diagnostic challenge in current practice. In recent years, several apparent tumor subtypes have emerged [4, 5]. On the one hand, behavior of eosinophilic renal cell tumors is in general highly favorable, and it is debatable whether these entities should be distinguished from the well-established oncocytoma and eosinophilic chromophobe RCC [3]. However, it appears that several of these have recognizable morphologies that correlate with distinct immunohistochemical and molecular features [4, 5]. For example, ESC RCC has reproducible morphology composed of solid nests of cells and cysts lined by cells with eosinophilic cytoplasm. Often the cells contain basophilic stippling of the cytoplasm, and positivity for keratin 20 is common, though sometimes focal or absent. Finally, these tumors have consistent alterations in the TSC genes, lending support to distinction from oncocytoma and chromophobe RCC, of which TSC gene alterations are present in only a minority of the latter [6–12]. ESC RCC has been included as an entity in the current WHO Classification of Tumors [6]; however, other candidate renal tumor types with eosinophilic cells are not yet included in the Classification, such as LOT and EVT, which would be classified under “other oncocytic tumors.” PRNRP would be classified under papillary RCC. As such, we sought to assess the current acceptance of emerging oncocytic tumor entities across an international group of genitourinary pathologists.

Participants in the survey noted encountering eosinophilic renal neoplasms relatively regularly, ranging from 5 to 20% of renal tumor specimens and sometimes as much as 50% of consultation specimens. Difficult to classify renal tumors were often noted as a monthly occurrence (48% of responses). For renal oncocytoma, there remains some uncertainty as to which features are acceptable for diagnosis, such as how much keratin 7 reactivity, binucleation, atypia, or involvement of structures (vessels or fat) remain compatible with the diagnosis [13]. In the current survey, we found that most pathologists would not render an outright diagnosis of oncocytoma in the biopsy setting (60%); however, there remain some genitourinary pathologists who would. This situation is debatable. On the one hand, it is difficult to know where the cutoff between oncocytoma and chromophobe RCC should be drawn, as prognosis for oncocytic neoplasms in general is highly favorable, so diagnosis in the biopsy setting is even more challenging, without access to the entire tumor. However, in other organs, there is not a precedent that one cannot diagnose, for example, tubular adenoma of the colon or ductal carcinoma in situ of the breast without a comment that invasive adenocarcinoma cannot be excluded due to sampling.

**Fig. 2:**
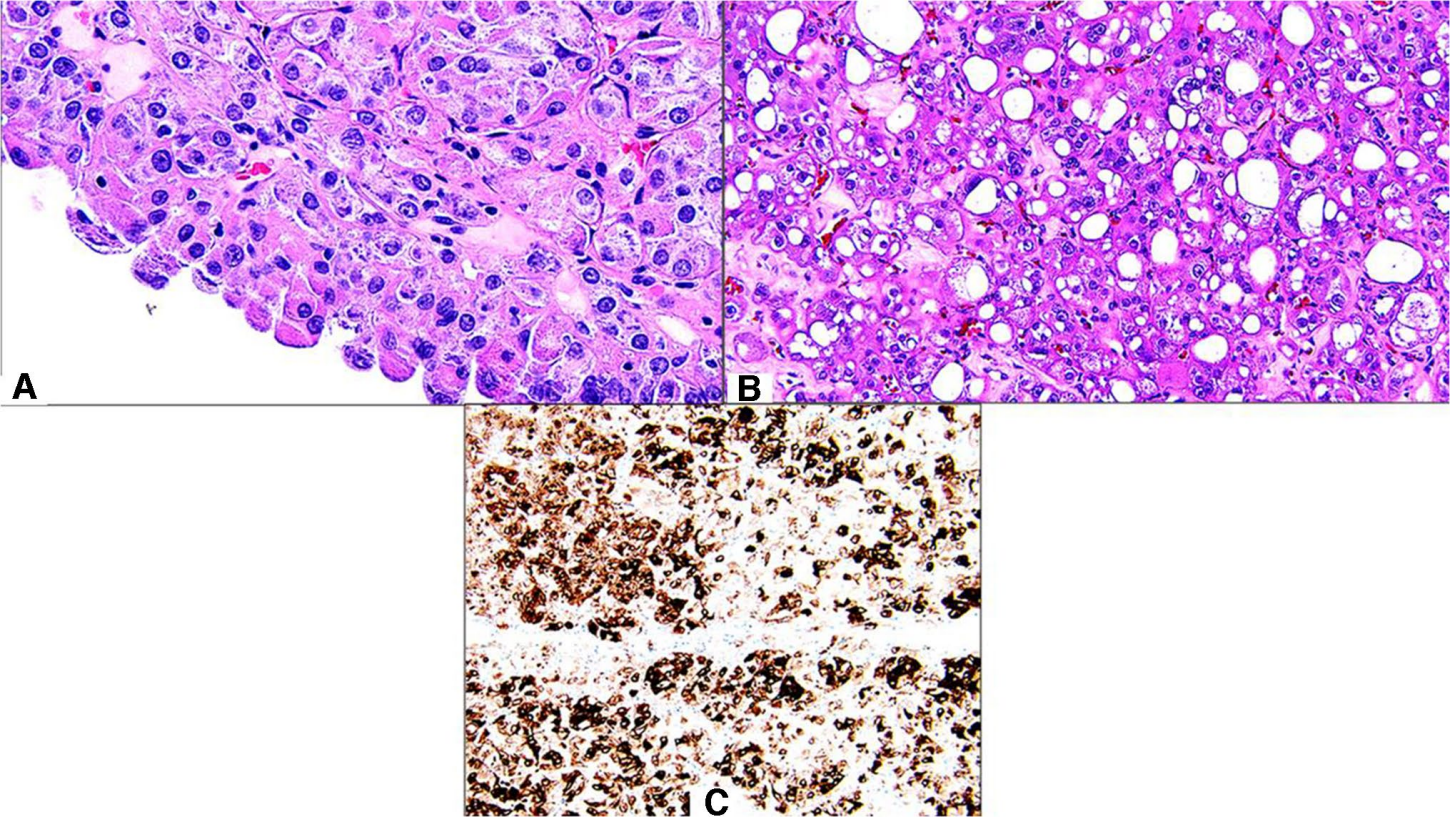
An example of eosinophilic solid and cystic renal cell carcinoma: (**A, B**) this tumor is characterized by an admixture of solid and cystic architecture, eosinophilic cytoplasm, with coarsely granular, basophilic cytoplasmic stippling, and focal to diffuse immunoreactivity for keratin 20 (**C**)

For the emerging renal tumor types, there was strongest support for ESC RCC as a distinct entity (70%), which is likely not surprising, since this has been designated as an entity in the current WHO Classification (Fig. 2) [6]. However, there remain some participants who felt that additional studies were needed, that this should be grouped with other entities, or (2%) that it is not a distinct entity at all. When considering grouping ESC RCC with other entities, a logical hypothesis would be to consider grouping the emerging entities with TSC/MTOR pathway alterations as a family. However, a limitation of such an approach is that there are differences in the morphology and immunohistochemistry of ESC RCC, LOT, and EVT that facilitate their discrimination from one another. Of these, only ESC RCC has been shown to definitively metastasize, also raising the possibility of behavioral differences. For LOT and EVT, the largest proportion of respondents making up approximately half (51% and 44%, respectively) indicated that there was not sufficient evidence for these tumors as distinct entities currently, but there would likely be in the future. In contrast, 27% and 29%, respectively, felt that there was sufficient evidence already. A limitation of the current study is that some time has passed since the survey was conducted, during which support for these entities may have changed. The survey window also just preceded the beta release of the WHO Urinary Tract text. Although many of the authors of the current study were also WHO authors, it is possible that awareness of preliminary WHO drafts or lack thereof also influenced the results. The largest proportion of respondents would use the new EVT nomenclature proposed for this entity by the Genitourinary Pathology Society (GUPS) consensus paper on emerging renal tumor types [5], whereas smaller numbers would still utilize prior names for this tumor type, such as RCC with eosinophilic vacuolated cytoplasm (16%) or high-grade oncocytic tumor (10%).

**Fig. 3:**
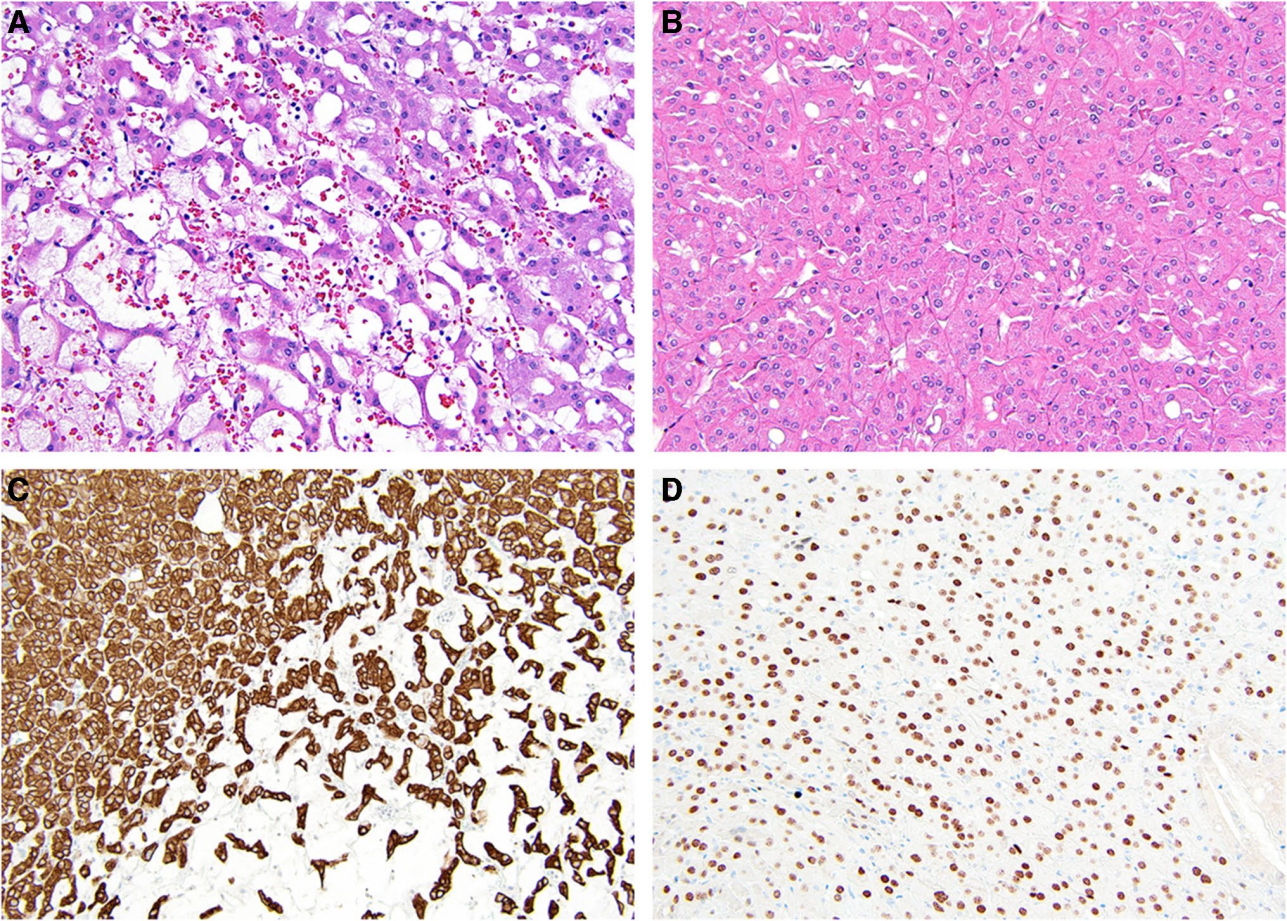
An example of low-grade oncocytic tumor:** A** loose cells interconnect with each other in an end-to-end distribution rather than round nests as seen in oncocytoma; **B** other areas show solid growth with round to oval nuclei, and a lack of “raisinoid” nuclei;** C** staining for keratin 7 is diffuse; **D** GATA3 is positive

In brief, LOT is noted to have a monomorphic pattern of eosinophilic cells with round, uniform nuclei (Fig. 3). Perinuclear clearing (“halo”) may or may not be present. In contrast to oncocytoma, which typically shows a very small percentage of cells positive for keratin 7 in a scattered distribution, LOT is diffusely positive for this marker, a feature that would have likely led to consideration as eosinophilic chromophobe RCC by some in the past. Likewise, contrasting to oncocytoma and chromophobe RCC, which are typically positive for KIT, LOT shows a negative reaction [4, 14–20]. Recently, it has been noted that GATA3 is also consistently positive in LOT [20]. A challenge with the nomenclature of this tumor type is that the term LOT may be perceived as a descriptive nonspecific diagnosis, similar to terminology such as “oncocytic renal neoplasm” often used in the biopsy setting when a definitive diagnosis cannot be made. One group has recently proposed the term “principle cell adenoma” [21] to highlight the presumptive line of differentiation of the tumor cells and use language that sounds like a more specific diagnosis.

EVT is composed of eosinophilic to pale cells with highly prominent nucleoli and one or more cytoplasmic vacuoles. There is often hyalinized stroma, and thick-walled blood vessels may be present. Despite the shared molecular pathogenesis with LOT, keratin 7 shows only rare positivity in EVT, more like an oncocytoma pattern [22–26]. KIT is variable but often positive. Cathepsin K may be positive, which overlaps the phenotype of translocation-associated RCC and other entities. In the current survey, the most common immunohistochemical markers used for eosinophilic renal tumors in general included keratin 7 (*n* = 61; 97%), KIT (*n* = 61; 97%), PAX8 (*n* = 46; 73%), SDHB (*n* = 45; 71%), CA9 (*n* = 40; 64%), AMACR (*n* = 37; 59%), keratin 20 (*n* = 36; 57%), FH (*n* = 36; 57%), melan A (*n* = 32; 51%), vimentin (*n* = 30; 48%), CD10 (*n* = 26; 41%), and cathepsin K (*n* = 25; 40%). However, since the survey was conducted, an emerging marker, GPNMB, appears to show promise for recognizing renal neoplasms with MITF family translocations and TSC/MTOR pathway alterations [27, 28], which was not assessed in this study.

A recent editorial (3) raised concern that these emerging tumor types may be clinically insignificant. Indeed, these entities appear to be largely favorable, and it is not clear that discrimination from eosinophilic chromophobe RCC would have clinical impact, as the entire spectrum from oncocytoma to LOT, EVT, and eosinophilic chromophobe RCC exhibits nonaggressive behavior. It also remains difficult to determine the exact cutoff between oncocytoma and eosinophilic chromophobe RCC, and aggressive behavior appears to occur rarely, if ever [13]. Some authors have suggested that regardless of specific diagnosis, active surveillance for oncocytic neoplasms diagnosed by renal mass biopsy is safe [29]. However, some possible areas of impact are that ESC RCC has been reported to metastasize [7], whereas the others (EVT and LOT) have not to date. Additionally, in view of the TSC/MTOR pathway alterations in these tumor types, it appears that a subset is associated with tuberous sclerosis complex [26, 30, 31]. This likely resembles the situation with the *VHL* gene, in which patients with germline mutation are prone to develop clear cell RCC, but patients without germline mutation also can develop clear cell RCC due to multiple genetic “hits” to *VHL*. Therefore, recognizing these tumors, especially when multiple or in young patients, may suggest an inherited syndrome in a subset.

Despite the recognition of genetic alterations in these tumor types, utilization of genetic testing in clinical practice to diagnose renal tumors still appears limited. Over half of the survey participants indicated that they use genetic tests rarely (*n* = 35; 56%). However, the most used tests include *TFE3* FISH (*n* = 45; 71%), *TFEB* FISH (*n* = 31; 49%), *TSC1* NGS (*n* = 21; 33%), *TSC2* NGS (*n* = 20; 32%), and *MTOR* NGS (*n* = 17; 27%).

**Fig. 4:**
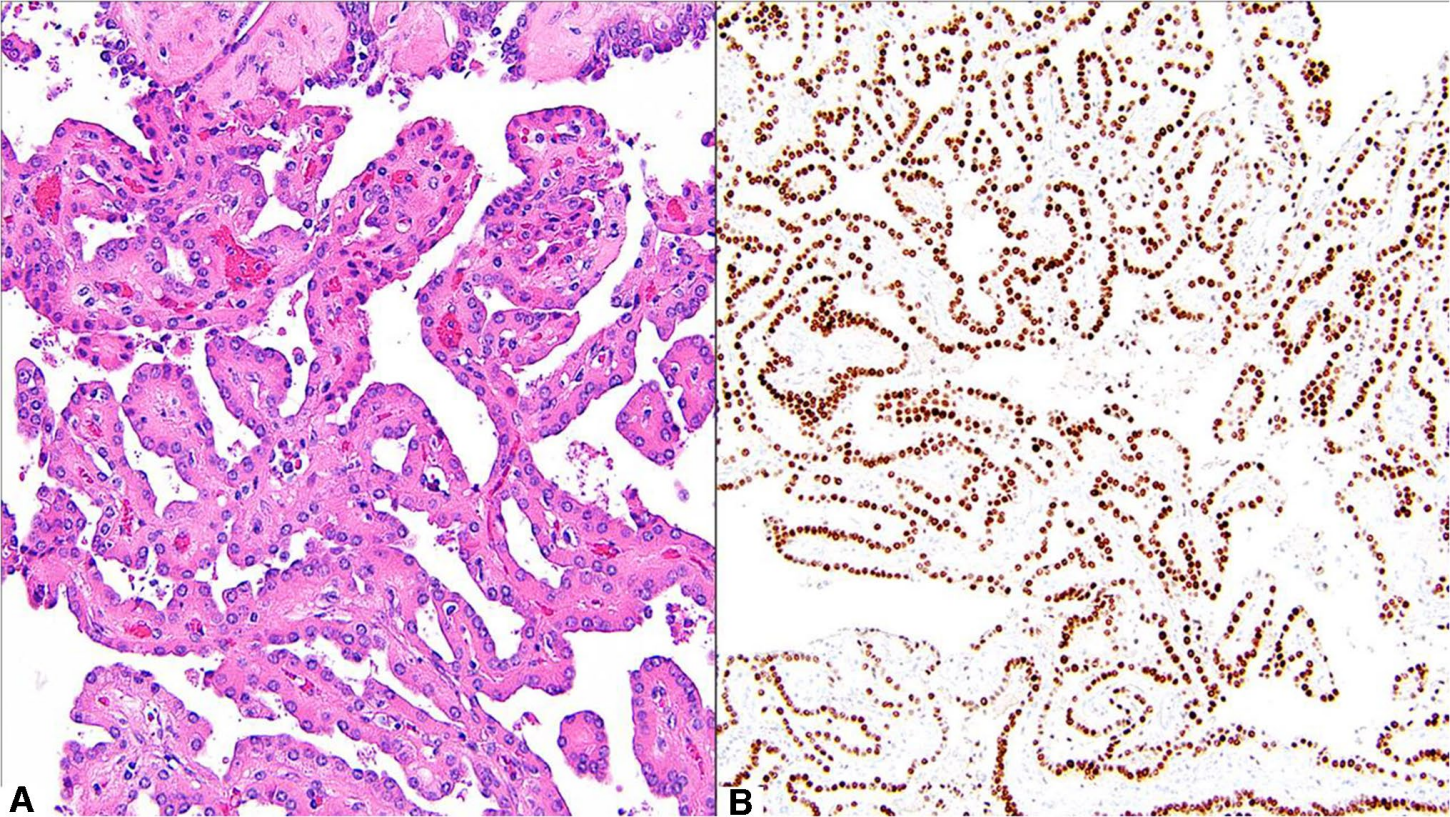
An example of papillary renal neoplasm with reverse polarity: **A** thin arborizing papillary architecture with hyalinized papillae. The lining cells are cuboidal with eosinophilic finely granular cytoplasm containing apically located low-grade nuclei opposite to the basement membrane; **B** the tumor exhibited strong and diffuse nuclear expression for GATA3

As another pattern of renal tumor with eosinophilic cells, we also queried perception of the tumor type now known as papillary renal neoplasm with reverse polarity (Fig. 4). This tumor type has been previously referred to as oncocytic papillary RCC or papillary RCC with oncocytic cells and nonoverlapping low-grade nuclei [32]. It was not clear that prior studies addressed a single homogenous diagnostic entity, and therefore in the past “oncocytic papillary RCC” had not gained traction as a specific diagnosis. However, recent scholarship has solidified a consistent morphology, immunohistochemical pattern, and genetic finding in this tumor type, coalescing largely around the name papillary renal neoplasm with reverse polarity [32–54]. Although tumors with papillary architecture may have eosinophilic cells that do not meet criteria for this entity [55], those with low-grade nuclei aligned toward the apex of the cell, coupled with immunohistochemical positivity for GATA3, frequent keratin 7, and negative vimentin, have a recurrent pattern of genetic alterations in the *KRAS* gene and favorable behavior. Although not currently included in the WHO Classification, this tumor is discussed under the section regarding papillary RCC. Only 38% of respondents felt there was sufficient evidence for consideration of this tumor type as a distinct entity now; however, 63% felt that there was either sufficient evidence now or would be in the future. Also, as noted previously, since time has passed after the recognition of this pattern, acceptance may have increased since the survey was conducted, as a number of additional studies have been published [36–47].

**Fig. 5:**
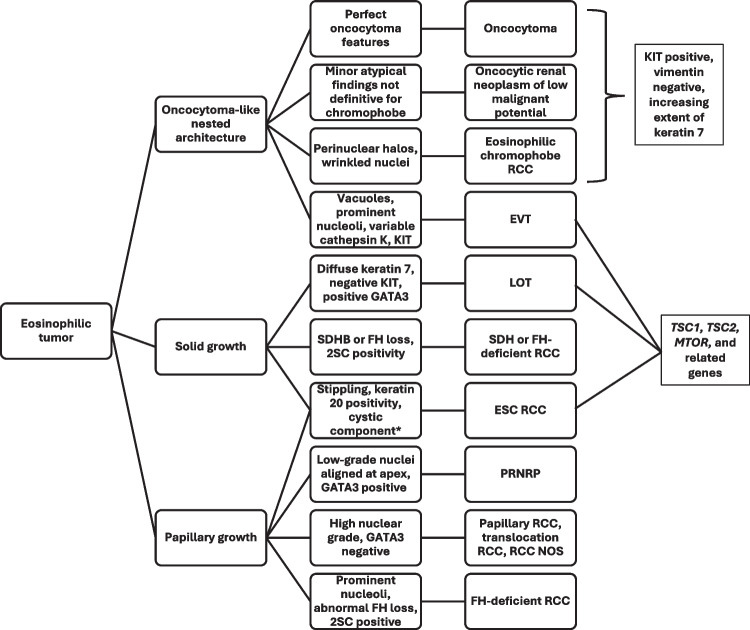
A flowchart shows a potential algorithm for discriminating eosinophilic renal tumors, with emphasis on emerging subtypes. Abbreviations: RCC, renal cell carcinoma; EVT, eosinophilic vacuolated tumor; LOT, low-grade oncocytic tumor; ESC, eosinophilic solid and cystic; PRNRP, papillary renal neoplasm with reverse polarity; NOS, not otherwise specified. *Not all features are present in every tumor

In summary, there remains incomplete acceptance of emerging eosinophilic renal tumor types in current practice; however, all of the emerging entities discussed in this survey appear to have strong promise for eventual acceptance, based on collapsing responses of those who felt that there was sufficient evidence for a distinct entity *currently* or *likely in the future*, to yield 63% for PRNRP and > 70% for both LOT and EVT. ESC RCC is most strongly accepted as a distinct entity, with 70% acknowledging sufficient evidence currently. This is reinforced by its inclusion in the current WHO Classification. Further study will be helpful to determine to what extent these diagnoses have clinical implications, since in general, eosinophilic renal tumors are typically highly favorable. Occurrence of a subset in the setting of tuberous sclerosis may be one clinical implication for a subset. A potential diagnostic algorithm for these entities is shown in Fig. 5.

## Supplementary Information

Below is the link to the electronic supplementary material.

Supplementary file1 (DOCX 24 KB)

## Data Availability

Data from this study are available from the corresponding author upon reasonable request.
