# Supplementary material for: Acceptance of emerging renal oncocytic neoplasms: a survey of urologic pathologists
Source: Virchows Arch. 2024 Sep 17;485(5):829–40. doi: 10.1007/s00428-024-03909-2 (PMC11564393; doi:10.1007/s00428-024-03909-2)
Supplement: Supplementary file 1 — Supplementary file1 (DOCX 24 KB) [file 428_2024_3909_MOESM1_ESM.docx]

**The Current State of Emerging Renal Oncocytic Neoplasms: A Survey of Urologic Pathologists.**

**1.Please mention your years of practice/How many years are you into practice?**

a.<5 years

b. 5-10 years

b. 10-20 years

c.   >20 years

**2.**      **Please mention your geographic location of practice/Where do you practice pathology?**

a. North America

b.      South America

c.       Asia

d.      Australia

e.       Africa

f.       Europe

**3**. **What percentage of tumors(approximately) at your institution would you considerto be renal cell carcinomas with eosinophilic features?**

**4. What percentage of the tumors (approximately) can you classify into a specific subtype from the above group?**

**_____________**

**5. How often do you encounter eosinophilic renal neoplasms with unusual histology that are difficult to classify into a specific subtype (either oncocytoma or chromophobe renal cell carcinoma)?**

a. Daily

b. Monthly

c. Every few months

d. Few times in a year

e. Yearly

f. Never

**6. A subset of renal tumors has recently been described as low-grade oncocytic tumor (keratin 7 positive, KIT negative). Would you consider this a distinct entity?**

a. Enough evidence for a distinct entity currently

b. Not currently but likely will have sufficient evidence eventually

c. Should be grouped together with one or more other emerging oncocytic entities

d. Uncertain

e. No

# 7. A subset of renal tumors has recently been described as eosinophilic vacuolated tumor (high-grade oncocytic tumor / renal cell carcinoma with eosinophilic vacuolated cytoplasm). Would you consider this a distinct entity?

a. Enough evidence for a distinct entity currently

b. Not currently but likely will have sufficient evidence eventually

c. Should be grouped together with one or more other emerging oncocytic entities

d. Uncertain

# e. No

# 8.A subset of renal tumors has recently been described as eosinophilic solid and cystic renal cell carcinoma. Would you consider this a distinct entity?

a. Enough evidence for a distinct entity currently

b. Not currently but likely will have sufficient evidence eventually

c. Should be grouped together with one or more other emerging oncocytic entities

d. Uncertain

# e. No

**9. How often do you encounter the recently described renal neoplasms with eosinophilic/oncocytic features (low-grade oncocytic tumor (LOT), eosinophilic vacuolated tumor (renal cell carcinoma with eosinophilic and vacuolated cytoplasm / high-grade oncocytic tumor), and eosinophilic, solid and cystic renal cell carcinoma (ESC) in your practice?**

a. Daily

b. Weekly

c. Few times a month

d. Monthly

e. Every few months

f. Few months a year

g. Yearly

h. Never

**10. Would you report an outright diagnosis of oncocytoma on a needle core biopsy?**

a. Yes

b. No

c. Other; with a comment

**11. When the morphology and immunohistochemical features of an oncocytic/eosinophilic neoplasm are conflicting/overlapping in a needle core biopsy which of the following diagnostic terminology you most often would use in your practice?**

a. Oncocytoma

b.Oncocytic neoplasm, favor oncocytoma pending complete excision

c. Oncocytic neoplasm, see note (explaining overlapping findings)

d. Oncocytic neoplasm, favor chromophobe carcinoma

e. Chromophobe carcinoma

**12**. **Oncocytoma needs to be differentiated from other oncocytic neoplasms because it is a benign tumor and a strict follow up is not mandatory?**

a. Yes

b. No

**13. Would you render a diagnosis of an oncocytoma in a tumor with classical oncocytoma morphology and diffuse positivity for CD117 in complete absence of CK7 staining?**

a. Yes

b. No

**14.Which of the following histochemical or immunohistochemical stains do you routinely or selectively use in your laboratory for diagnosis of oncocytic renal tumors (select all, and include only staining techniques that you use for diagnostic purposes but exclude those used only for research purposes or those which you have discontinued utilization)**

a.CK7 (Keratin 7)

b. CK20 (Keratin 20)

c. Vimentin

d. C-kit (CD117)

e. Cathepsin K

f. AMACR

g. CD 10

h. RCC antigen

i. PAX2

j. PAX8

k. E-cadherin

l. Ksp cadherin

m. Parvalbumin

n. Colloidal iron (Hales or modified Mowry colloidal iron)

o. HNF1β

p. CAIX

q. S100A1

r. MOC31

s. CD15

t. EpCAM

u. CD82

v. EMA

w. Galectin 3

x. Caveolin 1

y. MUC1

z. Glutathione S-transferase alpha

a1. Melan A

b1. SDH-B

c1. FH

d1. Others (please specify any other marker)

**15**. **Would you perform any immunohistochemistry in the diagnostic work up of every oncocytoma?**

a. Yes

b. No

**16. Which of following circumstances would warrant immunostaining in an oncocytoma?**

a. I always use immunohistochemistry before diagnosing oncocytoma.

b. Compact nesting pattern/solid

c. Mild nuclear membrane irregularity with subtle perinuclear clearing

d.Binucleation

e. Nuclear grooves

f. Fat invasion

g. Vascular invasion

h. Small cell features (oncoblastic)

i. None of the above

**17. Do you use the terminology low-grade oncocytic tumorwhen the tumor is diffusely positive for CK7/Keratin 7 and does not show any CD117 staining?**

a. Yes

b. No

c. Diagnose as oncocytoma or chromphobe RCC with comment

d. Other

**18.A renal tumor shows a mixture of eosinophilic papillary and non-papillary morphology without cystic areas, and diffuse positivity for CK20/Keratin 20. FH and SDH are retained within the tumor cells. How would you likely report such a tumor?**

a.Chromophobe renal cell carcinoma

b. Renal cell carcinoma, unclassified

c. Low-grade oncocytic tumor

d. Type 2 papillary renal cell carcinoma

e. Eosinophilic solid and cystic renal cell carcinoma

f. HLRCC associated renal cell carcinoma

**19.How often do you use Cathepsin K in the diagnostic work up of an eosinophilic renal epithelial neoplasm?**

a.Frequently (most eosinophilic neoplasms)

b. Sometimes (if a specific differential diagnosis is raised)

c. Rarely (only if highly suggestive of a specific entity)

d. Never

e. I might use it but not available in my practice/area

**20.How often do you use TFE3 immunohistochemistry in the diagnostic work up of an eosinophilic renal epithelial neoplasm?**

a.Frequently (most eosinophilic neoplasms)

b. Sometimes (if a specific differential diagnosis is raised)

c. Rarely (only if highly suggestive of a specific entity)

d. Never

e. I might use it but not available in my practice/area

**21**.**How often do you use TFE3 FISH/NGS in the diagnostic work up of an eosinophilic renal epithelial neoplasm?**

a. Frequently (most eosinophilic neoplasms)

b. Sometimes (if a specific differential diagnosis is raised)

c. Rarely (only if highly suggestive of a specific entity)

d. Never

e. I might use it but not available in my practice/area

**22**. **Which terminology would you use for a tumor fitting the description of the recently-described entity eosinophilic vacuolated tumor / high-grade oncocytic tumor / renal cell carcinoma with eosinophilic vacuolated cytoplasm?**

a. Eosinophilic vacuolated tumor (GUPS proposal)

b. High-grade oncocytic tumor (He et al Virchow Arch)

c. Renal cell carcinoma with eosinophilic vacuolated cytoplasm (Chen et al Am J Surg Pathol)

d. Borderline or hybrid oncocytic neoplasm

e. Chromophobe renal cell carcinoma

f. Unclassified renal cell carcinoma

g. Other

**23**.**Which of the following genetic tests do you use for diagnosis of oncocytic neoplasms (select all that apply)?**

a.Karyotype

b. Copy number assessment (FISH)

c. Copy number assessment (array)

d. *TSC1* sequencing

e. *TSC2* sequencing

f. *MTOR* sequencing

g. *VHL* sequencing

h. *BHD (FLCN)* sequencing

i. *TFE3* FISH

j. *TFEB* FISH

k. *TFE3* or *TFEB* sequencing

l. Other

**24.How often do you use genetic techniques such as those selected in the previous question to diagnose oncocytic neoplasms?**

a. Most or all oncocytic neoplasms

b. Frequently but less than half (25-50%)

c. 25% or less

d. Rarely

e. Never

**25.Would you ask for a familial screening for extrarenal neoplasms such as GIST, in a subset of oncocytic renal neoplasms?**

a. Yes

b. No

**26**. **Do you consider papillary renal neoplasm with reverse polarity / oncocytic papillary renal neoplasm with inverted nuclei to be a distinct entity?**

a. Enough evidence for a distinct entity currently

b. Not currently but likely will have sufficient evidence eventually

c. Should be grouped together with one or more other emerging oncocyticentities

d. Uncertain

# e. No

**27.Have you encountered low-grade oncocytic renal neoplasms that are deficient for fumarate hydratase (mimicking succinate dehydrogenase-deficient renal cell carcinoma), as described in Smith et al Histopathology?**

a. Yes

b. No

c. Other
